# The identification of cases of major hemorrhage during hospitalization in patients with acute leukemia using routinely recorded healthcare data

**DOI:** 10.1371/journal.pone.0200655

**Published:** 2018-08-15

**Authors:** Aukje L. Kreuger, Rutger A. Middelburg, Erik A. M. Beckers, Karen M. K. de Vooght, Jaap Jan Zwaginga, Jean-Louis H. Kerkhoffs, Johanna G. van der Bom

**Affiliations:** 1 Center for Clinical Transfusion Research, Sanquin Research, Leiden, the Netherlands; 2 Department of Clinical Epidemiology, Leiden University Medical Center, Leiden, the Netherlands; 3 Department of Hematology, Maastricht University Medical Center, Maastricht, the Netherlands; 4 Department of Clinical Chemistry and Haematology, University Medical Center Utrecht, Utrecht, the Netherlands; 5 Department of Immunohaematology and Blood Transfusion, Leiden University Medical Center, Leiden, the Netherlands; 6 Department of Hematology, Haga Hospital, Den Haag, the Netherlands; Mathematical Institute, HUNGARY

## Abstract

**Introduction:**

Electronic health care data offers the opportunity to study rare events, although detecting these events in large datasets remains difficult. We aimed to develop a model to identify leukemia patients with major hemorrhages within routinely recorded health records.

**Methods:**

The model was developed using routinely recorded health records of a cohort of leukemia patients admitted to an academic hospital in the Netherlands between June 2011 and December 2015. Major hemorrhage was assessed by chart review. The model comprised CT-brain, hemoglobin drop, and transfusion need within 24 hours for which the best discriminating cut off values were taken. External validation was performed within a cohort of two other academic hospitals.

**Results:**

The derivation cohort consisted of 255 patients, 10,638 hospitalization days, of which chart review was performed for 353 days. The incidence of major hemorrhage was 0.22 per 100 days in hospital. The model consisted of CT-brain (yes/no), hemoglobin drop of ≥0.8 g/dl and transfusion of ≥6 units. The C-statistic was 0.988 (CI 0.981–0.995). In the external validation cohort of 436 patients (19,188 days), the incidence of major hemorrhage was 0.46 per 100 hospitalization days and the C-statistic was 0.975 (CI 0.970–0.980). Presence of at least one indicator had a sensitivity of 100% (CI 95.8–100) and a specificity of 90.7% (CI 90.2–91.1). The number of days to screen to find one case decreased from 217.4 to 23.6.

**Interpretation:**

A model based on information on CT-brain, hemoglobin drop and need of transfusions can accurately identify cases of major hemorrhage within routinely recorded health records.

## Introduction

Electronic health care data are increasingly used for research purposes.[1–3] It offers the potential to investigate rare events and to obtain reliable estimates using large populations or specific subgroups with long follow-up time, while maintaining high external validity.[3–5]

Within the field of hematology, studies regarding bleeding could benefit from electronic health care data. Bleeding can be categorized according to the WHO criteria, a scale from 1 to 4, in which grade 1 indicates petechiae and grade 4 debilitating blood loss[6] Major hemorrhages (WHO grade 3–4) are clinically most relevant, but occur infrequently. To obtain sufficient power, many studies use a composite endpoint consisting of all bleeding events WHO grade ≥2.[7–10] However, it has been suggested that including WHO grade 2 bleedings in a composite outcome is not valid.[11] Instead, it would be preferable to include only hemorrhages WHO grade 3 and 4, although this would require large sample sizes.

Several algorithms have been developed to identify bleeding events from administrative data and these are mostly based on billing data or ICD codes.[12] The reliability of such an algorithm depends upon the quality of the administrative coding and regional and temporal variation exists.[13] In contrast to billing data and ICD codes, routinely recorded clinical data, like laboratory measurements, are more objective and could therefore potentially be used to improve the identification of bleeding events.[12] These data are easily obtainable and do not require any additional effort by clinicians. The aim of this study was to develop a model to identify patients with a high likelihood of major hemorrhage (WHO grade 3–4) within a database of routinely recorded clinical data of adult patients with acute leukemia without a detailed review of patient files.

## Methods

### Setting and population

The model was developed using routinely recorded clinical data of a cohort of adult patients with acute leukemia admitted to the Leiden University Medical Center in the Netherlands between June 2011 and December 2015. The model was externally validated within a cohort of adult acute leukemia patients admitted to the University Medical Center Utrecht or to the Maastricht University Medical Center between January 2010 and January 2016.

In all cohorts, patients were selected based on the ‘diagnosis treatment combination’ code (in Dutch ‘DBC, diagnose behandel combinatie’). The DBC code is a national system for the registration and reimbursement of health care activities.[14]. Patients with acute lymphatic or myeloid leukemia, or refractory anemia with excess blasts (RAEB) were included in this study (DBC codes 756, 761, and 762). The study protocol was approved by the Medical Ethical Committee of the Leiden University Medical Hospital, University Medical Center Utrecht, and Maastricht University Medical Center, and the scientific committee of the Center for Clinical Transfusion Research, Sanquin. All data were pseudonymized and the ethical committees waived the requirement for informed consent.

### Variables

Routinely recorded clinical data were extracted from the electronic health care system of the hospitals. Collected variables were age, gender, DBC codes, dates of hospitalizations, received blood products, hemoglobin measurements, and dates of CT-scans of the brain. Drop in hemoglobin per 24 hours was categorized into ≤0.8, >0.8 up to and including 1.6g/dl, >1.6 to 1.9 g/dl, >1.9 to 2.2 g/dl, >2.2 to 2.8 g/dl and >2.8 g/dl. Transfusion need was defined as total number of blood products per 24 hours, including red blood cells, platelets and plasma and categorized in ≤2, 3, 4, 5, and ≥6 blood products.

Information about bleeding was collected via chart review and classified according to the WHO Severity Grading System with the specifications as used in the PlaDo trial: grade 1 petechiae, grade 2 mild blood loss, grade 3 gross blood loss, grade 4 debilitating blood loss (S1 Table).[6, 15] Major hemorrhage, WHO grade 3 or 4, was taken as primary outcome. Secondary, all bleedings, regardless of WHO grade, were included.

### Sample

Chart review was performed for a sample of observation days during hospital admission, selected according to the following strategy. All eligible hospitalization days were first stratified by categories of hemoglobin drop and number of transfusions, and from each of these strata we aimed to include 20 days. Additionally, all days on which a CT-brain was performed were reviewed. To ensure no bleeding was missed due to patient or doctor’s delay, a time frame of one day before and one day after the selected date was reviewed. As a negative control, we selected 90 days on which maximal one blood product was transfused and the drop in hemoglobin was less than 0.8 g/dL. Sampling was performed without replacement and restricted to one day per hospital admission per indicator. Using this selection procedure, the sample was enriched with days with a potentially increased risk of bleeding. To adjust for this, the sample was weighted according to the prevalence of the indicators in the original cohort for all analyses and the calculation of the incidence of hemorrhage. With the final sample of 352 hospitalization days, we could establish a specificity of 96% with a precision of 2% and an alpha of 0.05, assuming an incidence of 0.5 cases per 100 hospitalization days.

### Development of the model

The results of the chart review were used as golden standard for the outcome of major hemorrhage. Drop in hemoglobin per 24 hours and transfusion need per 24 hours were taken as indicators for major blood loss and CT-brain during hospital stay as an indicator for potential intracranial hemorrhage. A logistic model was fitted to predict the risk of major hemorrhage. For all indicators the sensitivity, specificity, negative and positive predictive value, and C-statistic were calculated. For the continuous predictors, the cut-off value with the best discriminative capacity was entered into the model. Discrimination is the ability to separate patients who had a hemorrhage from those who had not and is quantified by the C-statistic. A C-statistic of 1.0 denotes perfect discrimination and a C-statistic of 0.5 represents discrimination equivalent to random chance.[16] The model was internally validated using bootstrap resampling with 100 repetitions. Performance of the model was expressed by the sensitivity, specificity, negative and positive predictive value with exact binomial 95% confidence intervals and summarized by the C-statistic. In addition, we calculated the number of days needed to screen to detect one case of major hemorrhage for all predicted risks.

### External validation

The model was externally validated in a cohort of leukemia patients from two other academic hospitals in the Netherlands. The same methods as in the derivation cohort were used to select the patients and extract the required data. The predicted risk of major hemorrhage was calculated using the model. Chart review was performed for all days with a predicted risk >0.01, 100 random control days with a predicted risk of 0.006, and 100 control days with a predicted risk of 0.0002. Discriminative capacity was quantified by sensitivity, specificity, negative and positive predictive value, and the C-statistic. A calibration plot was made to illustrate the agreement between expected risks and observed outcomes. Perfect calibration is characterized by a line with an intercept of 0 and a slope of 1.[17]

## Results

### Study population

The derivation cohort consisted of 255 patients, 10,638 observation days, compromising 1,319 hospital admissions. The median length of admission was one day (interquartile range (IQR) 1–23), reflecting the large number of day admissions. Thirty-eight percent of admissions was longer than one day, median 27 days (IQR 16–35). The median age of the patients was 56.9 (IQR 44.3–65.4), most were men (60.4%) and the majority was diagnosed with acute myeloid leukemia (74.1%) (Table 1). Chart review was performed for a random sample of 353 hospitalization days (149 patients). The final sample contained more days with certain characteristics than would be expected solely based on the sampling scheme, since transfusion need and drop in hemoglobin are correlated (Table 1).

**Table 1:** Patient characteristics.

	Complete derivation cohort	Sample derivation cohort
**Patients**	255	149
**Male gender (%)**	154 (60.4)	87 (58.4)
**Age in years, median (IQR)**	56.9 (44.3–65.4)	58.4 (44.9–67.2)
**Diagnosis**		
AML (%)	189 (74.1)	113 (75.8)
RAEB (%)	20 (7.8)	11 (7.4)
ALL (%)	46 (18.0)	25 (16.8)
**Hospital admissions (n)**	1319	265
**Length of hospital stay, median (IQR)**	1 (1–23)	25 (2–35)
**Observation days**	10,638	353
CT-scan (%)	75 (0.7)	75 (21.3)
Hemoglobin drop		
>0.8 to 1.6g/dl (%)	572 (5.4)	42 (11.9)
>1.6 to 1.9 g/dl (%)	29 (0.3)	20 (5.7)
≥1.9 to 2.2 g/dl (%)	49 (0.5)	22 (6.2)
≥2.2 to 2.8 g/dl (%)	18 (0.2)	18 (5.1)
≥2.8 g/dl (%)	13 (0.1)	13 (3.7)
Transfusion need		
2 products (%)	1,270 (11.9)	50 (14.2)
3 products (%)	1,126 (10.6)	43 (12.2)
4 products (%)	418 (3.9)	40 (11.3)
5 products (%)	156 (1.5)	31 (8.8)
≥ 6 products (%)	136 (1.3)	31 (8.8)
Control (%)	7216 (67.8)	90 (25.5)

Within the sample, 19 cases of major hemorrhage were found, corresponding to 16 unique patients. Of these, ten hemorrhages were intracranial, four gastro-intestinal, three following an invasive procedure, one pulmonary and one vaginal. None of the hemorrhages occurred during a day admission. Extrapolated to the complete cohort of 255 patients, 6.3% of patients experienced major hemorrhage, corresponding to an incidence of 0.22 per 100 hospitalization days. Including all grades of severity, 43 patients suffered from a bleeding event on 59 different days. Extrapolated to the complete cohort, the incidence of any hemorrhage was 8.4 per 100 hospitalization days.

### Derivation cohort

**Fig 1:**
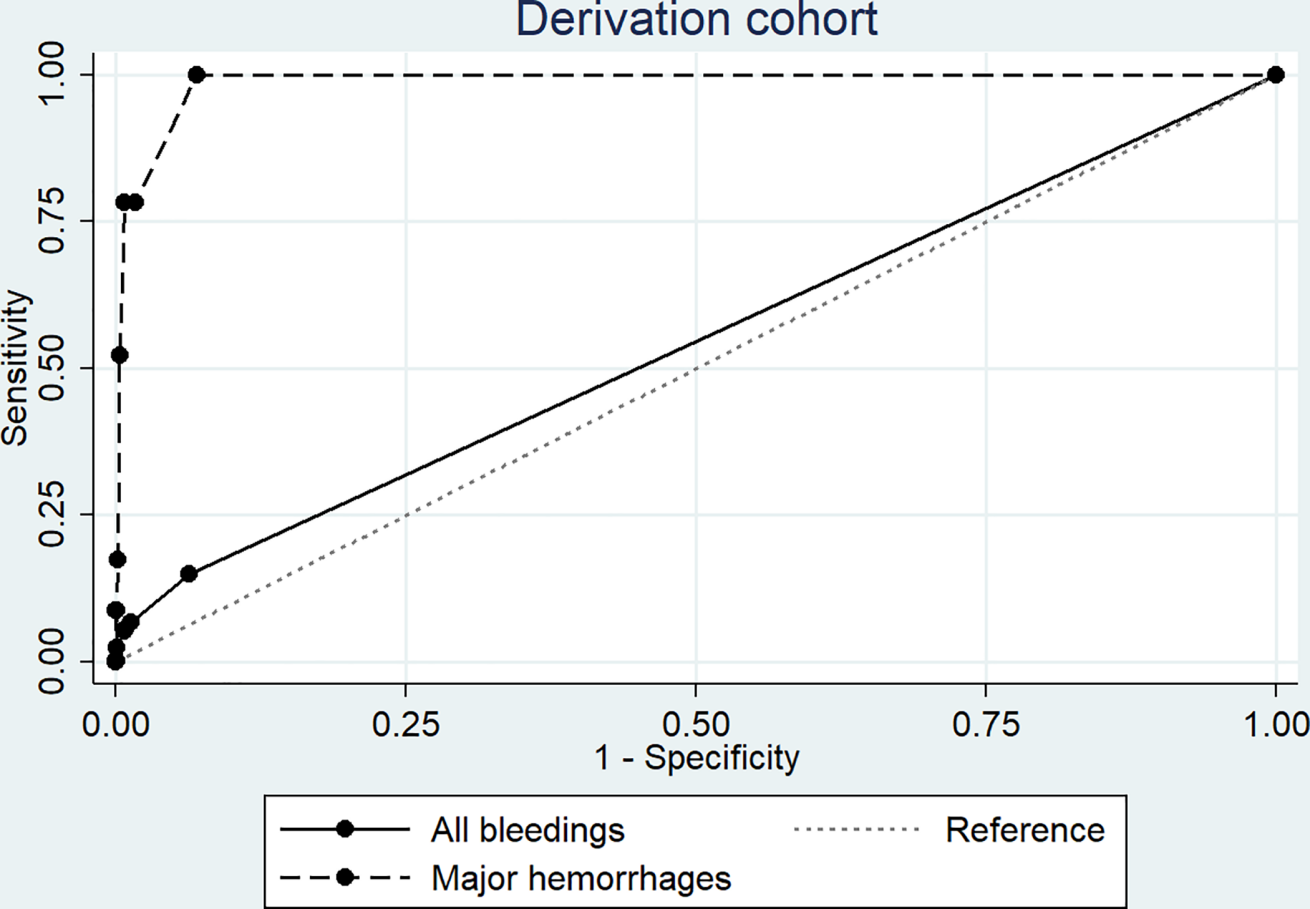
ROC curve of the model in the derivation cohort. AUC for major hemorrhages was 0.988 (0.981: 0.995), for bleedings of all severity 0.545 (0.533: 0.557). The depicted results are derived from the sample and extrapolated to the entire cohort.

Univariable analysis revealed that a hemoglobin drop of at least 0.8 g/dl and the need of six or more transfusions had the best discriminative capacity for major hemorrhage and for bleedings of all grades (Table 2, S2 Table). Combined with the CT- brain (yes/no), the complete model had a C-statistic of 0.988 (confidence interval (CI) 0.981 to 0.995) for major hemorrhage and of 0.545 (CI 0.533 to 0.557) for all bleedings (Fig 1). The coefficients of the model are depicted in S3 Table. CT- brain or a combination of any of two indicators corresponded to a predicted risk of ≥0.02, with a sensitivity of 78.3% (CI 56.3 to 92.5) and a specificity of 99.2% (CI 99.1 to 99.4) (Table 3). When at least one indicator is present (predicted risk ≥0.006), the sensitivity was 100% (CI 85.2 to 100) with a specificity of 93.1% (CI 92.6 to 93.5) (Table 3). With an incidence of 0.22 per 100 hospitalization days, 454.5 days have to be screened to detect one case. This is reduced to 5.5 days when a predicted risk of ≥0.02 is taken as cut off (Table 3).

**Table 2:** Univariable predictive capacity for major hemorrhage for CT-scan of the brain and several cut-off values of hemoglobin drop and transfusion need.

Variables	Sensitivity in % (CI)	Specificity in % (CI)	Positive predictive value in % (CI)	Negative predictive value in % (CI)	C-statistic (CI)
CT-scan brain	43.5 (23.2; 65.5)	99.4 (99.2; 99.5)	13.3 (6.6; 23.2)	99.9 (99.8; 99.9)	0.714 (0.611; 0.818)
Hemoglobin drop					
>0.8 g/dl	73.9 (51.6; 89.8)	94.5 (94.0; 94.9)	2.9 (1.7; 4.5)	99.9 (99.9; 100)	0.842 (0.750; 0.934)
≥1.6 g/dl	47.8 (26.8; 69.4)	99.2 (99.0; 99.4)	11.8 (6.1; 20.2)	99.9 (99.8; 99.9)	0.735 (0.631; 0.840)
≥2.0 g/dl	34.8 (16.4; 57.3)	99.4 (99.2; 99.5)	11.1 (4.9; 20.7)	99.9 (99.8; 99.9)	0.671 (0.571; 0.770)
≥2.4 g/dl	26.1 (10.2; 48.4)	99.8 (99.6; 99.8)	19.4 (7.5; 37.5)	99.8 (99.7; 99.9)	0.629 (0.537; 0.721)
≥2.8 g/dl	21.7 (7.5; 43.7)	99.9 (99.8; 100)	38.5 (13.9; 68.4)	99.8 (99.7; 99.9)	0.608 (0.522;0.694)
Transfusion need					
2 products	13.0 (2.8; 33.6)	88.0 (87.4; 88.7)	0.2 (0.05; 0.7)	99.8 (99.7; 99.9)	0.505 (0.435; 0.576)
3 products	4.4 (0.1; 21.9)	89.3 (88.7; 89.9)	0.1 (0.0; 0.5)	99.8 (99.6; 99.9)	0.468 (0.426; 0.511)
4 products	26.1 (10.2; 48.4)	96.6 (96.2; 96.9)	1.7 (0.6; 3.6)	99.8 (99.7; 99.9)	0.613 (0.522; 0.705)
5 products	4.4 (0.1; 21.9)	98.6 (98.4; 98.8)	0.7 (0.0; 3.7)	99.8 (99.7; 99.9)	0.515 (0.472; 0.557)
≥ 6 products	43.5 (23.2; 65.5)	98.9 (98.7; 99.1)	8.1 (4.0; 14.4)	99.9 (99.8; 99.9)	0.712 (0.608; 0.816)

**Table 3:** Characteristics and performance of the model in the derivation cohort.

Predicted risk*	CT†	Hb†	Tx†	Sensitivity in % (CI)	Specificity in % (CI)	Positive predictive value in % (CI)	Negative predictive value in % (CI)	Days needed to screen ‡	False negatives
**All**	0	0	0	100 (85.2; 100)	0 (0; 0.04)	0.2 (0.1; 0.3)	N/A§	454.5	0
**≥0.006**	0	+	0	100 (85.2; 100)	93.1 (92.6; 93.5)	3.1 (2.0; 4.6)	100 (100; 100)	34.7	0
**≥0.013**	0	0	+	78.3 (56.3; 92.5)	98.3 (98.1; 98.6)	9.3 (5.6; 14.3)	99.9 (99.9; 100)	11.0	5
**≥0.022**	+	0	0	78.3 (56.3; 92.5)	99.2 (99.1; 99.4)	18.4 (11.3; 27.5)	99.9 (99.9; 100)	5.5	5
**≥0.250**	0	+	+	52.2 (30.6; 73.2)	99.7 (99.6; 99.8)	27.9 (15.3; 43.7)	99.9 (99.8; 99.9)	3.6	11
**≥0.362**	+	+	0	17.5 (5.0; 38.8)	99.9 (99.8; 99.9)	20.0 (5.7; 43.7)	99.8 (99.7; 99.9)	5.1	19
**≥0.538**	+	0	+	8.7 (1.1; 28.0)	100 (99.9; 100)	50.0 (6.8; 93.2)	99.8 (99.7; 99.9)	2.0	21
**≥0.967**	+	+	+	8.7 (1.1; 28.0)	100 (100; 100)	100 (15.8; 100)	99.8 (99.7; 99.9)	1.0	21

The sample was reweighted according to the distribution of the indicators in the complete cohort. The total number of events in reweighted dataset was 23.* The predicted risks include the risk for a given risk factor or larger risks (the lines below)

†CT: CT scan brain, Hb: hemoglobin, Tx: transfusion.

+ indicates presence and 0 indicates absence of the indicator

‡ Calculated with an incidence of 0.22 per 100 days, which was the incidence in the extrapolated cohort.

§ N/A not applicable, negative predicted value can’t be calculated when all days are screened.

### Validation cohort

The external validation total cohort consisted of 436 patients, 19,188 hospitalization days, compromising 1,276 hospital admissions. The median length of admission was 17 days (IQR 2–32.5). In contrast to the hospital of the derivation cohort, day admissions were differently coded and therefore not included in the database. The median age of the patients was 57.7 year (IQR 46.0–65.5), 58.7% were men and 74.5% were diagnosed with acute myeloid leukemia (Table 4). The patient characteristics stratified by hospital are depicted in S4 Table. Chart review was performed for 599 hospitalization days (294 patients). For 17 days (9 patients) no information about bleeding could be retrieved from the patient files. These days were excluded from all analyses. Within the remaining 582 days (291 patients), 42 patients experienced major hemorrhage on 52 different days. Extrapolated to the complete cohort, this corresponded to an incidence of 0.46 per 100 hospitalization days. Assuming that all major hemorrhages were detected by using this model, 9.6% of the patients experienced major hemorrhage in the complete cohort. Seventeen were intracranial, seventeen gastro-intestinal, six urogenital, four followed an invasive procedure, three hemorrhages derived from the spleen, three patients had an epistaxis requiring a red blood cell transfusion, one patient had a pleural hemorrhage and one had a retina bleeding event with visual impairment.

**Table 4:** Baseline characteristics validation cohort.

	Validation cohort	Sample validation cohort
Patients	436	294
Male gender (%)	256 (58.7)	174 (59.2)
Age in years, median (IQR)	57.7 (46.0–65.5)	56.7 (40.5–65.4)
Diagnosis		
AML (%)	325 (74.5)	216 (73.5)
RAEB (%)	28 (6.4)	21 (7.1)
ALL (%)	83 (19.0)	55 (18.7)
Hospital admissions (n)	1,276	458
Length of hospital stay, median (IQR)	17 (2–32.5)	27 (10–37)
Observation days	19,188	599
CT-scan (%)	110 (0.57)	110 (18.4)
Hemoglobin drop		
>0.8 to 1.6g/dl (%)	1,293 (6.7)	203 (33.9)
>1.6 to 1.9 g/dl (%)	103 (0.5)	14 (2.3)
≥1.9 to 2.2 g/dl (%)	145 (0.8)	25 (4.2)
≥2.2 to 2.8 g/dl (%)	89 (0.5)	11 (1.8)
≥2.8 g/dl (%)	45 (0.2)	11 (1.8)
Transfusion need		
2 products (%)	1,159 (60)	81 (13.5)
3 products (%)	1,040 (5.4)	50 (8.4)
4 products (%)	656 (3.4)	14 (2.3)
5 products (%)	147 (0.8)	7 (1.2)
≥ 6 products (%)	51 (0.3)	92 (15.4)
Control (%)	56 (0.3)	400 (66.8)

**Fig 2:**
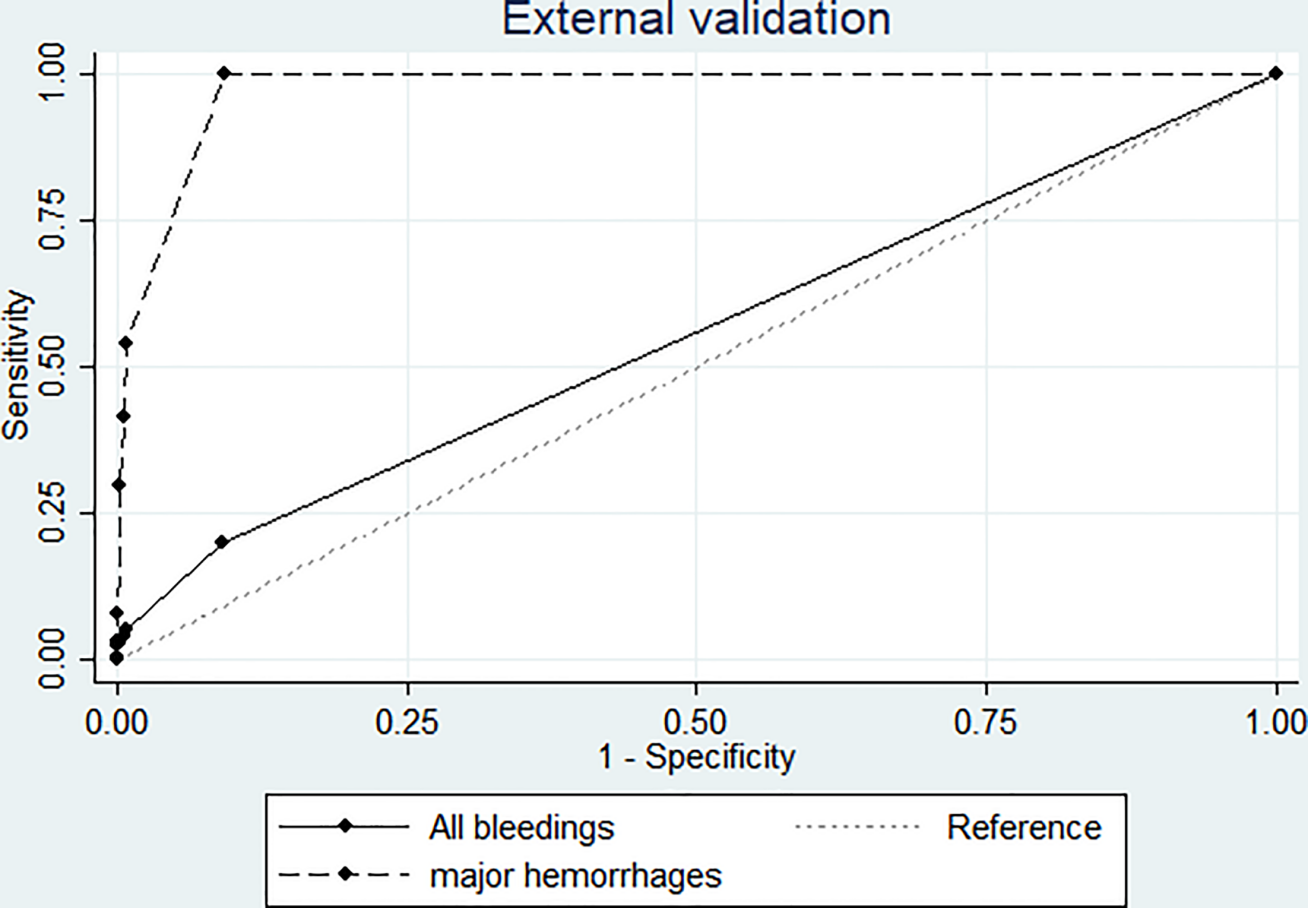
ROC curve for major hemorrhages and all bleedings in the external validation cohort. AUC for major hemorrhages was 0.975 (0.970: 0.980), for bleedings of all severity 0.557 (0.544: 0.569). The depicted results are derived from the sample and extrapolated to the entire cohort.

For a predicted risk of ≥0.02, the sensitivity of the model was 41.4% (CI 30.9 to 52.4), the specificity 99.4% (CI 99.3 to 99.5), and the days needed to screen 4.2. When at least one indicator was present (predicted risk ≥0.006) the sensitivity was 100% (CI 95.8 to 100), the specificity 90.7% (CI 90.2 to 91.1) and 23.6 days had to be screened to detect one case of major hemorrhage (Table 5 and S5 Table). The C-statistic of the model was 0.975 (CI 0.970;980) (Fig 2). Calibration of the model is shown in S1 Fig.

**Table 5:** Performance of the model in the external validation cohort.

Predicted risk	Sensitivity in % (CI)	Specificity in % (CI)	Positive predictive value in % (CI)	Negative predictive value in % (CI)	Days needed to screen*	False negatives
**All**	100 (95.8; 100)	0 (0; 0.02)	0.5 (0.4; 0.6)	N/A†	217.4	0
**≥0.006**	100 (95.8; 100)	90.7 (90.2; 91.1)	4.7 (3.8; 5.7)	100 (100; 100)	23.6	0
**≥0.013**	54.0 (43.0; 64.8)	99.2 (99.1; 99.3)	24.4 (18.5; 31.0)	99.8 (99.7; 99.8)	4.2	40
**≥0.022**	41.4 (30.9; 52.4)	99.4 (99.3;99.5)	24.3 (17.7; 32.1)	99.7 (99.6; 99.8)	4.2	51
**≥0.250**	29.9 (20.5; 40.6)	99.8 (99.7; 99.9)	41.9 (29.5; 55.2)	99.7 (99.6; 99.8)	2.4	61
**≥0.362**	8.1 (3.3; 15.9)	99.9 (99.9; 99.9)	29.2 (12.6; 51.1)	99.6 (99.5; 99.7)	3.5	80
**≥0.538**	3.5 (0.7; 9.8)	100 (100; 100)	100 (29.2; 100)	99.6 (99.5; 99.6)	1	84
**≥0.967**	2.3 (0.3; 8.1)	100 (100; 100)	100 (15.8; 100)	99.6 (99.4; 99.6)	1	85

The sample was reweighted according to the distribution of the indicators in the complete cohort. The total number of events in the reweighted dataset was 87.

* Calculated with an incidence of 0.46 per 100 days, which was the incidence in the extrapolated cohort.

† N/A not applicable, negative predicted value can’t be calculated when all days are screened.

Including all grades of severity, 65 patients suffered from a bleeding event on 83 different days. This corresponded to an incidence of 5.5 bleedings per 100 hospitalization days, or 2.4 bleedings per patient in the complete cohort. The C-statistic of the model for all bleedings was 0.557 (CI 0.544; 0.569) (Fig 2).

## Discussion

Routinely recorded data can be used to accurately identify cases of major hemorrhages, WHO grade 3 and 4, among patients with acute leukemia. A model based on drop in hemoglobin ≥0.8 g/dL, the need of ≥6 transfusions and CT-brain allows the capture of cases with major hemorrhages in large datasets over a long follow-up period while minimizing costs and effort. The model has poor discriminative capacity for bleedings of all grades of severity.

Cases identified with this model can be used as an outcome regarding studies investigating risk factors for bleeding in large populations or to identify cases for a case control study. The average incidence in all cohorts combined was 0.37 per 100 hospitalization days. This implies that 270 days have to be screened to find one case of major hemorrhage. When at least one of the indicators is present, the days to screen is limited to 34.7 to 23.1 days, without missing a single case. This could even be reduced to only 11 to 4.2 days by choosing a higher cut off risk, although with this strategy 40 of 87 (45.9%) cases will be missed. These are predominantly renal, gastrointestinal, and splenic hemorrhages, whereas all cases with intracranial bleeding will still be detected.

An advantage of routinely collected data is that it offers the opportunity to include larger populations which maximizes the generalizability. Additionally, patients in trials are mostly selected using rigorous in- and exclusion criteria which cannot be extrapolated to general practice [3]. A drawback of routinely collected data is that these are not collected for research purposes and therefore potentially more at risk for errors and missing data [18, 19]. The accuracy and completeness of these data has been demonstrated by linking 99% of fatal events of the West of Scotland Coronary Prevention Study (WOSCOPS) trial to routinely collected ICD codes [20, 21]. In addition, the incidence in our sample is comparable with the incidences reported in literature [11, 22, 23]. In the external validation cohort, we detected major hemorrhage among 9.6% of the patients, corresponding to an incidence of 0.46 per 100 days. A trial of 600 leukemia patients reported an incidence of 0.05 per 100 observation days.[23] In an observational study, the incidence was 5 out of 68 patients (7.8%) and in another trial this was 28 out of 255 patients (11%) [11, 22].

In the current study, major hemorrhage was not reported in a standardized way and patients were not stringently observed. Instead, we used proxies for major blood loss and intracranial bleed. Limitation of this approach is that cases with retinal bleed with visual impairment (WHO grade 4) will be missed. In addition, patients have to survive long enough after start of hemorrhage to reach the threshold of hemoglobin drop or transfusion need, or a CT-scan. Therefore the model could underestimate the true incidence of major hemorrhage. However, we assume this does not outweigh the benefits of including all patients leading to a considerable increase in sample size.

Algorithms are often based on coding sets used in specific datasets, like the ICD codes. These are prone to changes in coding or medical practice and regional and temporal variation exists.[24] In contrast to these algorithms, we included variables that are easily accessible and less prone to variation. Calibration of the model in the external validation was imperfect. However, this model is not aimed to predict risks, but primarily to discriminate. Discriminative capacity of the model was very good in the derivation cohort as well as in the external validation cohort, which confirms the overall generalizability of this model.

In conclusion, we developed and validated a model based on routinely collected clinical data to reliably identify patients with major hemorrhage. This model will have particular significance for researchers and blood services who aim to investigate major hemorrhage among hematological patients with sufficient sample size, by limiting the number of days to screen.

## Supporting information

S1 Table WHO bleeding score, with specifications as used in the PlaDo trial. (DOCX)

S2 Table Predictive capacity for CT-scan of the brain and several cut-off values of hemoglobin drop and transfusion need for bleeding of all severity. (DOCX)

S3 Table Beta’s of the model. (DOCX)

S4 Table Patients characteristics of the external validation cohort, stratified by hospital. (DOCX)

S5 Table Performance of the model in the external validation cohort stratified by hospital. (DOCX)

S1 Fig Calibration plot external validation. (DOCX)
