# Supplementary material for: The identification of cases of major hemorrhage during hospitalization in patients with acute leukemia using routinely recorded healthcare data
Source: PLoS One. 2018 Aug 15;13(8):e0200655. doi: 10.1371/journal.pone.0200655 (PMC6093651; doi:10.1371/journal.pone.0200655)
Supplement: S1 Table — (DOCX) [file pone.0200655.s001.docx]

**Online supplements**

**S1 Table 1. WHO bleeding score, with specifications as used in the PlaDo trial.^1^**

|  | **Grade 1** | **Grade 2** |
| --- | --- | --- |
| **Oral and nasal** | - Oropharyngeal bleeding or epistaxis, total duration ≤30 minutes in 24 hours - Petechiae of oral mucosa | - Oropharyngeal bleeding or epistaxis, total duration >30 minutes in 24 hours |
| **Skin, soft tissue, musculoskeletal** | - Petechiae - Purpura ≤1 inch diameter - Spontaneous hematomas in soft tissue or muscle >1 inch | - Purpura > 1 inchi diameter - Spontaneous hematomas in deeper tissue - Joint bleeding |
| **Gastrointestinal** | - Positive stool occult blood test | - Melanotic stool - Hematochezia - Hematemesis |
| **Genitourinary** | - Microscopic Hb/RBC without red urine - Abnormal vaginal bleeding with spotting | - Gross visible hematuria - Abnormal vagainal bleeding, more than spotting |
| **Pulmonary** |  | - Hemoptysis - Blood in broncho-pulmonary lavage |
| **Body cavity** |  | - Visible blood in body cavity fluid |
| **Central nervous system** |  | - Retinal bleeding without visual impairment - Lumbar puncture with >5RBC on microscopic analysis and no traumatic tap, no symptoms, no visible red color |
| **Invasive sites** |  | - Bleeding at invasive sites, active oozing >1 hours in 24 hours |

**Grade 3:**

- Any bleeding requiring RBC transfusion over routine transfusion need, specifically related to treatment of bleeding within 24 hours of onset of bleeding.
- Any bleeding associated with moderate hemodynamic instability (hypotension >30mmHg fall, or 30% decrease in systolic or diastolic blood pressure) and requiring RBC transfusion over routine transfusion need.
- Grossly bloody body cavity fluids and organ dysfunction with symptoms and need to intervene.
- Lumbar puncture with visible red color, in absence of symptoms and non-traumatic tap.

**Grade 4:**

- Fatal bleeding
- Any bleeding associated with severe hemodynamic instability (hypotension >50 mmHg or >50% decrease in systolic or diastolic blood pressure with tachycardia (increase heart rate >20% for 20 minutes) and requiring RBC transfusion.
- Retinal bleeding with visual impairment
- CNS symptoms with non-traumatic bloody lumbar puncture
- CNS bleeding on imaging study with or without dysfunction

1) Slichter SJ, Kaufman RM, Assmann SF, McCullough J, Triulzi DJ, Strauss RG, et al. Dose of prophylactic platelet transfusions and prevention of hemorrhage. The New England journal of medicine. 2010;362(7):600-13.
