# Supplementary material for: The identification of cases of major hemorrhage during hospitalization in patients with acute leukemia using routinely recorded healthcare data
Source: PLoS One. 2018 Aug 15;13(8):e0200655. doi: 10.1371/journal.pone.0200655 (PMC6093651; doi:10.1371/journal.pone.0200655)
Supplement: S2 Table — (DOCX) [file pone.0200655.s002.docx]

| **Variables** | | **Sensitivity (CI)** | **Specificity (CI)** | **Positive predictive value**  **(CI)** | **Negative predictive value**  **(CI)** | **C-statistic (CI)** |
| --- | --- | --- | --- | --- | --- | --- |
| **CT-scan brain** | | 1.8 (1.0; 2.9) | 99.4 (99.2; 99.6) | 22.2 (13.3; 33.6) | 91.7 (91.1; 92.2) | 0.506 (0.502; 511) |
| **Hemoglobin drop** | | | | | | |
| >0.8 g/dl | 11.1 (9.1; 13.4) | 94.9 (94.4; 95.3) | 16.6 (13.7; 19.9) | 92.1 (91.5; 92.6) | 0.530 (0.519; 0.541) |  |
| ≥1.6 g/dl | 2.7 (1.8; 4.0) | 99.3 (99.1; 99.5) | 26.4 (17.7; 36.7) | 91.7 (91.2; 92.3) | 0.510 (0.505; 0.516) |  |
| ≥2.0 g/dl | 2.2 (1.3; 3.3) | 99.5 (99.3; 99.6) | 26.8 (16.9; 38.6) | 91.7 (91.2; 92.2) | 0.508 (0.503; 0.513) |  |
| ≥2.4 g/dl | 1.0 (0.5;1.9) | 99.8 (99.7; 99.9) | 30.0 (14.7; 49.4) | 91.7 (91.1; 92.2) | 0.504 (0.501; 0.507) |  |
| ≥2.8 g/dl | 0.7 (0.3; 1.5) | 99.9 (99.8; 100) | 46.2 (19.2; 74.9) | 91.6 (91.1; 92.2) | 0.503 (0.500; 0.506) |  |
| **Transfusion need** | | | | | | |
| 2 products | 9.9 (8.1; 12.1) | 87.9 (87.2; 88.5) | 7.0 (5.7; 8.6) | 91.4 (90.8; 92.0) | 0.489 (0.479; 0.500) |  |
| 3 products | 6.0 (4.5; 7.8) | 88.9 (88.3; 89.5) | 4.7 (3.6; 6.2) | 91.2 (90.6; 91.7) | 0.475 (0.466; 0.483) |  |
| 4 products | 6.5 (4.9; 8.3) | 96.8 (96.4; 97.2) | 15.7 (12.1; 19.9) | 91.9 (91.3; 92.4) | 0.516 (0.508; 0.525) |  |
| 5 products | 3.3 (2.2; 4.7) | 98.8 (98.6; 99.0) | 20.4 (14.1; 28.0) | 91.8 (91.2; 92.3) | 0.511 (0.505; 0.517) |  |
| ≥ 6 products | 5.2 (3.8; 6.9) | 99.3 (99.1; 99.4) | 39.7 (30.7; 49.2) | 91.9 (91.4; 92.5) | 0.522 (0.515; 0.530) |  |

**S2 Table 1. Predictive capacity for CT-scan of the brain and several cut-off values of hemoglobin drop and transfusion need for bleeding of all severity.**
