# Supplementary material for: The identification of cases of major hemorrhage during hospitalization in patients with acute leukemia using routinely recorded healthcare data
Source: PLoS One. 2018 Aug 15;13(8):e0200655. doi: 10.1371/journal.pone.0200655 (PMC6093651; doi:10.1371/journal.pone.0200655)
Supplement: S3 Table — (DOCX) [file pone.0200655.s003.docx]

**S3 Table 1. Beta’s of the model**

| **Variable** | **Major bleeding, β(CI)** | **All bleeding, β(CI)** |
| --- | --- | --- |
| Constant (α) | -8.28 (-9.05; -7.5) | -2.50 (-2.58; -2.42) |
| CT brain (yes/no) | 4.48 (3.22; 5.74) | 0.90 (0.28; 1.52) |
| >0.8 g/dl drop in hemoglobin | 3.23 (2.06; 4.40) | 0.73 (1.49; 2.28) |
| ≥6 transfusions | 3.95 (2.88; 5.02) | 1.89 (1.49; 2.28) |
