# Supplementary material for: The identification of cases of major hemorrhage during hospitalization in patients with acute leukemia using routinely recorded healthcare data
Source: PLoS One. 2018 Aug 15;13(8):e0200655. doi: 10.1371/journal.pone.0200655 (PMC6093651; doi:10.1371/journal.pone.0200655)
Supplement: S4 Table — (DOCX) [file pone.0200655.s004.docx]

**S4 Table 1. Patients characteristics of the external validation cohort, stratified by hospital.**

|  | | | **Validation cohort A** | **Sample validation cohort A** | **Validation cohort B** | **Sample validation cohort B** |
| --- | --- | --- | --- | --- | --- | --- |
| **Patients** | | | 221 | 148 | 215 | 146 |
| **Male gender (%)** | | | 118 (53.4) | 78 (52.7) | 138 (64.2) | 96 (65.8) |
| **Age in years, median (IQR)** | | | 57.2 (40.2-65.7) | 56.8 (40.2-65.5) | 59.6 (49.6-66.8) | 59.7 (49.9-65.4) |
| **Diagnosis** | | |  |  |  |  |
| AML (%) | | 160 (72.4) | 108 (73.0) | 165 (76.8) | 110 (75.3) |  |
| RAEB (%) | | 20 (9.1) | 15 (10.1) | 8 (3.7) | 6 (4.2) |  |
| ALL (%) | | 41 (18.6) | 25 (16.9) | 42 (19.5) | 30 (20.5) |  |
| **Hospital admissions (n)** | | | 670 | 219 | 600 | 239 |
| **Length of hospital stay, median (IQR)** | | | 10 (1-29) | 27 (1-37) | 21.5 (6-39) | 28 (10-46) |
| **Observation days** | | | 6,519 | 271 | 12,669 | 328 |
| CT-scan (%) | | 37 (0.6) | 37 (13.7) | 73 (0.6) | 73 (22.2) |  |
| Hemoglobin drop | |  |  |  |  |  |
| >0.8 to 1.6g/dl (%) | 458 (7.0) | 96 (35.4) | 835 (6.6) | 107 (32.6) |  |  |
| >1.6 to 1.9 g/dl (%) | 35 (0.5) | 5 (1.9) | 68 (0.5) | 9 (2.7) |  |  |
| ≥1.9 to 2.2 g/dl (%) | 48 (0.7) | 15 (5.5) | 97 (0.8) | 10 (3.1) |  |  |
| ≥2.2 to 2.8 g/dl (%) | 42 (0.6) | 6 (2.2) | 47 (0.4) | 5 (1.5) |  |  |
| ≥2.8 g/dl (%) | 20 (0.3) | 7 (2.6) | 25 (0.2) | 4 (1.2) |  |  |
| Transfusion need | |  |  |  |  |  |
| 2 products (%) | 964 (14.8) | 45 (16.6) | 1,040 (8.2) | 36 (11.0) |  |  |
| 3 products (%) | 566 (8.7) | 30 (11.1) | 656 (5.2) | 20 (6.1) |  |  |
| 4 products (%) | 129 (2.0) | 9 (3.3) | 147 (1.2) | 5 (1.5) |  |  |
| 5 products (%) | 30 (0.5) | 4 (1.5) | 51 (0.4) | 3 (0.9) |  |  |
| ≥ 6 products (%) | 36 (0.6) | 36 (13.3) | 56 (0.4) | 56 (17.1) |  |  |
| Control* (%) | | 6,448 (98.9) | 200 (73.8) | 12,541 (99.0) | 200 (60.9) |  |

* all days with a predicted risk <0.01 could be selected as control day
