# Supplementary material for: The identification of cases of major hemorrhage during hospitalization in patients with acute leukemia using routinely recorded healthcare data
Source: PLoS One. 2018 Aug 15;13(8):e0200655. doi: 10.1371/journal.pone.0200655 (PMC6093651; doi:10.1371/journal.pone.0200655)
Supplement: S5 Table — (DOCX) [file pone.0200655.s005.docx]

**S5 Table 1. Performance of the model in the external validation cohort stratified by hospital.**

The sample was reweighted according to the distribution of the indicators in the complete cohort. The total number of cases in reweighted dataset for hospital A was 47 and for hospital B this was 40. The percentages are given and in brackets the 95% confidence interval.

| Predicted risk | Sensitivity (%) | Specificity (%) | Positive predictive value (%) | Negative predictive value (%) | Days needed to screen† (n) | False negatives  (n) |
| --- | --- | --- | --- | --- | --- | --- |
| Hospital A | | | | | | |
| All | 100 (92.5; 100) | 0 (0; 0.1) | 0.74 (0.55; 0.99) | N/A* | 135.2 | 0 |
| ≥0.006 | 100 (92.5; 100) | 90.1 (89.3; 90.8) | 7.0 (5.2; 9.2) | 100 (99.9; 100) | 16.0 | 0 |
| ≥0.013 | 61.7 (46.4; 75.5) | 99.3 (99.1; 99.5) | 40.9 (29.3; 53.2) | 99.7 (99.5; 99.8) | 2.5 | 18 |
| ≥0.022 | 55.3 (40.1; 69.8) | 99.5 (99.3; 99.7) | 44.8 (31.7; 58.5) | 99.7 (99.5; 99.8) | 2.3 | 21 |
| ≥0.250 | 44.7 (30.2; 59.9) | 99.9 (99.7; 99.9) | 70.0 (50.6; 85.3) | 99.6 (99.4; 99.7) | 1.4 | 26 |
| ≥0.362 | 8.5 (2.4; 20.4) | 99.9 (99.8; 100) | 44.4 (13.7; 78.8) | 99.3 (99.1; 99.5) | 2.3 | 43 |
| ≥0.538 | 4.2 (0.5; 14.5) | 100 (99.9; 100) | 100 (15.8; 100) | 99.3 (99.1; 99.5) | 1 | 45 |
| ≥0.967 | 2.1 (0.1; 11.3) | 100 (99.9; 100) | 100 (2.5; 100) | 99.3 (99.0; 99.5) | 1 | 46 |
| Hospital B | | | | | |  |
| All | 100 (91.2; 100) | 0 (0; 0.03) | 0.3 (0.2; 0.4) | N/A* | 322.6 |  |
| ≥0.006 | 100 (91.2; 100) | 90.9 (90.4; 91.4) | 3.4 (2.4; 4.6) | 100 (100; 100) | 32.4 | 0 |
| ≥0.013 | 45.0 (29.3; 61.5) | 99.2 (99.0; 99.3) | 14.8 (9.0; 22.3) | 99.8 (99.7; 99.9) | 6.9 | 20 |
| ≥0.022 | 25.0 (12.7; 41.2) | 99.4 (99.2; 99.5) | 11.1 (5.5; 19.5) | 99.8 (99.7; 99.8) | 9.3 | 28 |
| ≥0.250 | 12.5 (4.2; 26.8) | 99.8 (99.7; 99.9) | 15.6 (5.3; 32.8) | 99.7 (99.6; 99.8) | 6.5 | 33 |
| ≥0.362 | 7.5 (1.6; 20.4) | 99.9 (99.8; 100) | 20.0 (4.3; 48.1) | 99.7 (99.6; 99.8) | 5.1 | 35 |
| ≥0.538 | 7.5 (1.6; 20.4) | 99.9 (99.8; 100) | 20.0 (4.3; 48.1) | 99.7 (99.6; 99.8) | 5.1 | 35 |
| ≥0.967 | 2.5 (0.1; 13.2) | 100 (100; 100) | 100 (2.5; 100) | 99.7 (99.6; 99.8) | 1 | 37 |

* N/A not applicable, negative predicted value can’t be calculated when all days are screened.

† Calculated with an incidence of 0.74 per 100 days for hospital A and 0.31 per 100 days for hospital B.
