# Supplementary material for: The identification of cases of major hemorrhage during hospitalization in patients with acute leukemia using routinely recorded healthcare data
Source: PLoS One. 2018 Aug 15;13(8):e0200655. doi: 10.1371/journal.pone.0200655 (PMC6093651; doi:10.1371/journal.pone.0200655)
Supplement: S1 Fig — (DOCX) [file pone.0200655.s006.docx]

**S1 Fig 1. Calibration plot external validation**
